# TRPV1 Activation Attenuates High-Salt Diet-Induced Cardiac Hypertrophy and Fibrosis through PPAR-*δ* Upregulation

**DOI:** 10.1155/2014/491963

**Published:** 2014-07-24

**Authors:** Feng Gao, Yi Liang, Xiang Wang, Zongshi Lu, Li Li, Shanjun Zhu, Daoyan Liu, Zhencheng Yan, Zhiming Zhu

**Affiliations:** ^1^Department of Hypertension and Endocrinology, Center for Hypertension and Metabolic Diseases, Chongqing Institute of Hypertension, Daping Hospital, Third Military Medical University, Chongqing 400042, China; ^2^Department of Ultrasound, Daping Hospital, Third Military Medical University, Chongqing 400042, China; ^3^Department of Cardiovascular Diseases, Xinqiao Hospital, Third Military Medical University, Chongqing 400037, China

## Abstract

High-salt diet-induced cardiac hypertrophy and fibrosis are associated with increased reactive oxygen species production. Transient receptor potential vanilloid type 1 (TRPV1), a specific receptor for capsaicin, exerts a protective role in cardiac remodeling that resulted from myocardial infarction, and peroxisome proliferation-activated receptors *δ* (PPAR-*δ*) play an important role in metabolic myocardium remodeling. However, it remains unknown whether activation of TRPV1 could alleviate cardiac hypertrophy and fibrosis and the effect of cross-talk between TRPV1 and PPAR-*δ* on suppressing high-salt diet-generated oxidative stress. In this study, high-salt diet-induced cardiac hypertrophy and fibrosis are characterized by significant enhancement of HW/BW%, LVEDD, and LVESD, decreased FS and EF, and increased collagen deposition. These alterations were associated with downregulation of PPAR-*δ*, UCP2 expression, upregulation of iNOS production, and increased oxidative/nitrotyrosine stress. These adverse effects of long-term high-salt diet were attenuated by chronic treatment with capsaicin. However, this effect of capsaicin was absent in TRPV1^−/−^ mice on a high-salt diet. Our finding suggests that chronic dietary capsaicin consumption attenuates long-term high-salt diet-induced cardiac hypertrophy and fibrosis. This benefit effect is likely to be caused by TRPV1 mediated upregulation of PPAR-*δ* expression.

## 1. Introduction

Left ventricular hypertrophy is an adaptive response of the heart to hypertension or cardiovascular disease. In addition, it is a risk factor for the development of arrhythmias and diastolic dysfunction and progression of congestive heart failure [1]. Increasing evidence from clinical and epidemiology studies suggests that high-salt diet causes left ventricular hypertrophy independent of its effect on hypertension [2, 3]. The reactive oxygen species (ROS) production is elevated in Dahl salt-sensitive rat fed with high salt, which play a critical role in myocardium remodeling and heart failure due, in part, to its harmful effect on myocardial contractile dysfunction and structural damage [4, 5].

Peroxisome proliferation-activated receptors *δ* (PPAR-*δ*) are known to be expressed ubiquitously, and the predominant PPAR subtype of cardiac cells. PPAR-*δ* initially acts as a mediator in myocardial energy metabolism, and, recently, PPAR-*δ* is found to mitigate cardiac hypertrophy through inhibiting NF-*κ*B activation [6]. Besides, PPAR-*δ* activation can inhibit the oxidative stress-induced apoptosis in cardiomyocytes [7]. Transient receptor potential vanilloid type 1 (TRPV1) is a nonselective cation channel that can be activated by its specific agonist, capsaicin, a pungent compound in hot chili peppers [8]. Our previous studies showed that TRPV1 activation by dietary capsaicin improved endothelium-dependent vasorelaxation and regulated blood pressure in rats [9] and prevented high-salt diet-induced hypertension in mice [10, 11]. Huang and his colleagues reported that TRPV1 plays a protective role in cardiac remodeling that resulted from myocardial infarction [12]. However, it remains unknown whether chronic activation of TRPV1 via dietary capsaicin could attenuate cardiac hypertrophy induced by long-term high-salt diet. Based on these, we hypothesize that TRPV1 activation by capsaicin might prevent cardiac damage induced by oxidative stress through PPAR-*δ* upregulation.

## 2. Materials and Methods

### 2.1. Animal Preparation

Male C57BL/6J wild-type (WT) and TRPV1-null (TRPV1^−/−^) mice (Jackson Lab, MN, USA) were housed in a pathogen-free animal facility and allowed to have water and food ad libitum. All of the animals were subject to controlled temperature (22 ± 1°C) and lighting (lights on 6:00 AM to 6:00 PM). Mice were randomly grouped and fed with a normal-salt diet (NS, 0.5% NaCl by weight), high-salt diet (HS, 8% NaCl by weight), or high-salt plus capsaicin diet (HS + Cap, 8% NaCl, and 0.01% capsaicin by weight) for 1 year. All of the experimental procedures were performed in accordance with protocols approved by the Institutional Animal Care and Research Advisory Committee.

### 2.2. Cell Culture

The embryonic rat-heart-derived H9c2 cells (Cell Bank, Chinese Academy of Sciences, Shanghai, China) were maintained in growth medium composed of DMEM supplemented with 10% fetal bovine serum. H9c2 cells were plated at a density of 5,000 cells/cm^2^ and allowed to proliferate in growth medium. Medium was changed every 2 days. After incubation at 37°C in humid air (5% CO_2_ and 95% O_2_), for near confluence, the H9c2 cells were then deprived of serum and incubated for another 24 h before treatment.

### 2.3. Immunofluorescence Staining

H9c2 cells and cardiac tissue slides from WT mice were fixed with 10% formalin at room temperature for 60 min and then bathed in a 2% hydrogen peroxide methanol solution for 30 min. The cells were incubated with TRPV1-specific antibodies (Alomone, Israel) overnight at 4°C and incubated with fluorescent dye-labeled secondary antibodies (ZSGB-BIO, China) at room temperature for 30 min. Images were obtained with a TE2000-U Nikon eclipse microscope and analyzed with NIS-Elements imaging software (Nikon, Japan).

### 2.4. Intracellular Free Calcium Measurement

H9c2 cells grown on glass cover slips were loaded with Fura-2 (2 *μ*mol/L) and 0.025% Pluronic F-127 in a physiological saline solution for 40 min at room temperature in the dark. [Ca^2+^]_*i*_ was stained with Fura-2/AM and measured using the PTI Fluorescence Master Systems (Photon Technology International, Birmingham, NJ, USA). Fluorescence was measured at 510 nm emission, with excitation wavelengths of 340 and 380 nm, at baseline and after stimulation with capsaicin either with or without pretreatment with iRTX (1 *μ*M) for 5 min.

### 2.5. Echocardiographic Assessment

Echocardiography of all animals was performed at the end of 1 year using a Philips IE33 (Philips Medical Systems, Andover, MA) and a L12-5 MHz phased array probe. All measurements were made in accordance with the conventions of the American Society of Echocardiography and were conducted by the same investigator who was blinded to the experimental groups. For the procedure, animals were anaesthetized with 1% pentobarbital sodium (80 mg/kg body weight, i.p.), and left ventricular end-diastolic and end-systolic diameters (LVEDD and LVESD, resp.) and LV posterior wall thickness (PWT) were measured from midpapillary, short-axis images obtained by M-mode echocardiography. The percentage of LV fractional shortening (% FS), an index of contractile function, was calculated as FS (%) = [(LVEDD – LVESD)/LVEDD]. LV mass was calculated using a standard cube formula, which assumes a spherical LV geometry according to the formula: LV mass = 1.04 [(IVSd + LVEDD + LVPWd)^3^ − (LVEDd)^3^], where 1.04 is the specific gravity of the myocardium. The derived LV mass was normalized for body weight (LVW/BW) [13].

### 2.6. Histological Analysis

Histological analysis was performed using standard techniques. Excised hearts were rinsed in PBS, fixed in 4% paraformaldehyde for 16 h at 4°C, and dehydrated in a series of ethanol washes. Samples were subsequently cleared in xylene and mounted in paraffin. Sections of 5 *μ*M in thickness were cut and stained with hematoxylin and eosin to analyze tissue morphology or with Masson trichrome staining to analyze collagen content and fibrosis [14].

### 2.7. Western Blotting Analysis

Cells were treated with capsaicin (100 nM) in the presence or absence of iRTX (1 *μ*M) for 24 h before total protein was extracted. Tissues were homogenized and cells were lysed in high-salt extraction buffer (0.5 mol/L Tris, 1% NP-40, 1% Triton X-100, 1 g/L sodium dodecyl sulfate, 1.5 mol/L NaCl, 0.2 mol/L EDTA, and 0.01 mol/L EGTA) and 0.2 mmol/L protease inhibitor, placed at −20°C for 20 min, and centrifuged at 12 000 ×g at 4°C for 20 min to remove insoluble material. Protein concentration was determined using a DC protein assay kit (Bio-Rad, Hercules, CA, USA). A total of 50 *μ*g portions of the protein were resolved on SDS-polyacrylamide gels and electroblotted onto polyvinylidene difluoride membranes. After transfer, the membranes were blocked for 4 h at room temperature in blocking buffer (Bio-Rad). Next, the membranes were incubated overnight at 4°C with antibodies for TRPV1 (Alomone, Israel), PPAR-*δ* (Cell Signaling, USA), UCP2 (Santa Cruz, USA), iNOS (Cell Signaling, USA), and GAPDH (Santa Cruz, USA). After incubation with secondary antibodies (ZSGB-BIO, China) at room temperature for 2 h, the proteins were detected with enhanced chemiluminescence and quantified using a Gel Doc 2000 Imager (Bio-Rad). Protein expression was normalized to the internal control GAPDH.

### 2.8. Immunohistochemistry for Nitrotyrosine

Freshly isolated left ventricle was embedded in tissue-freezing compound (OCT Tissue Tek, Fisher Scientific INC., NY, USA), and the specimens were cut into 5 *μ*m sections on cover slides. Immunohistochemistry for 3-nitrotyrosine was performed with rabbit (Millipore, UK) polyclonal antibodies (1 : 1000), using diaminobenzidine as the chromogen and nuclear counterstaining with hematoxylin. Images were obtained with a TE2000-U Nikon eclipse microscope and analyzed with NIS-Elements imaging software (Nikon, Japan) [15].

### 2.9. Statistical Analysis

Data are expressed as the mean ± SEM. Statistical differences between groups were assessed by Student's *t*-test or one-way analysis of variance with Bonferroni's multiple comparison post hoc tests, as appropriate. Two-sided *P* values below 0.05 were considered statistically significant.

## 3. Results

### 3.1. TRPV1 Characterization in Cardiac Muscles and Cardiomyocytes

**Figure 1:**
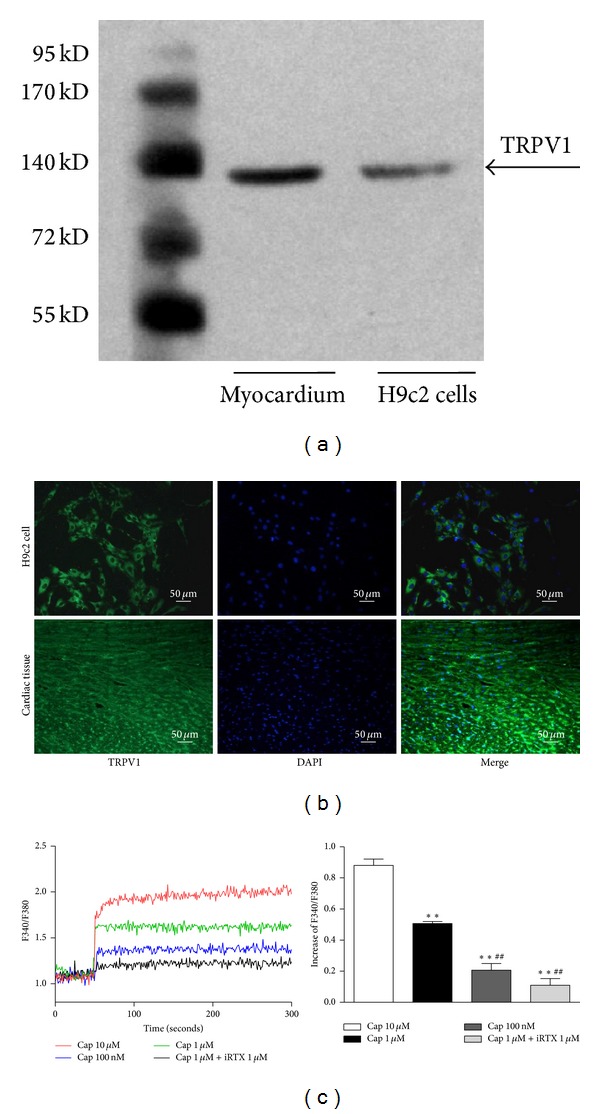
Characterization of TRPV1 in myocardium and H9c2 cells. (a) TRPV1 protein level was detected in myocardium and H9c2 cells. (b) TRPV1 localization in H9c2 cells and cardiac tissues of WT mice was shown with immunofluorescence. TRPV1 was stained in green and the nucleus was stained using DAPI (blue color). Images are representative of three separate experiments. The scale bar denotes 50 *μ*m. (c) Fura-2-loaded H9c2 cells were stimulated by increasing the concentration of capsaicin (0.1–10 *μ*mol/L) in the presence or absence of TRPV1 antagonist iRTX; values were expressed as the mean ± SEM from six separate experiments. Cap: capsaicin. **P < 0.01 versus Cap (10 *μ*mol/L) and ^##^ *P* < 0.01 versus Cap (1 *μ*mol/L).

To characterize TRPV1 in cardiac muscles and H9c2 cells, TRPV1 expression was detected by western blotting in freshly isolated left ventricular from WT mice and cultured H9c2 cells. The immunoblots confirmed that the molecular mass of TRPV1 is 95 KDa and the antibodies identified TRPV1 in both left ventricular from WT mice and H9c2 cells, as shown in Figure 1(a). Immunofluorescence staining shows that TRPV1 was located in both the H9c2 cells and cardiac tissues of WT mice (Figure 1(b)). To determine the function of TRPV1 channels in cardiomyocytes, we examined the intracellular free calcium concentration ([Ca^2+^]_*i*_). Acute capsaicin stimulation caused a marked increase of [Ca^2+^]_*i*_ in cultured cardiomyocytes (Figure 1(c)). Inhibition of TRPV1 by a specific blockade named 5′-iodo-resiniferatoxin (iRTX) prevented the capsaicin-induced [Ca^2+^]_*i*_ increase. These results indicated that functional TRPV1 is present in H9c2 cells and mouse cardiac muscles.

### 3.2. Dietary Capsaicin Improves Cardiac Function in WT Mice on Long-Term High-Salt Diet through TRPV1 Activation

**Figure 2:**
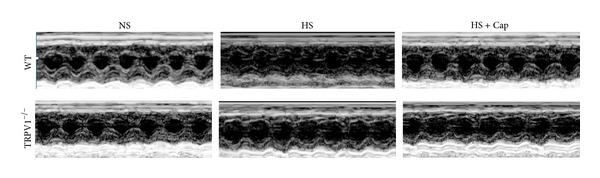
Representative echocardiography from wild-type (WT) and TRPV1-null (TRPV1^−/−^) mice on a normal diet (NS), a high-salt diet (HS), or a high-salt plus capsaicin diet (HS + Cap) for 1 year.

As reported in Table 1 and exemplified with M-mode tracings in Figure 2, left ventricular end-diastolic diameter (LVEDD) was significantly increased in both WT and TRPV1^−/−^ mice on a high-salt diet compared with their respective control (normal-salt fed) groups (*P* < 0.01 or *P* < 0.05). Chronic dietary capsaicin significantly prevented the increase of LVEDD in WT mice on a high-salt diet (*P* < 0.05). However, this effect of capsaicin was absent in TRPV1^−/−^ mice on a high-salt diet (*P* > 0.05). Similarly, other echocardiographic parameters such as fractional shortening (FS), left ventricular end-systolic diameter (LVESD), and left ventricular posterior wall (LVPW) have shown the same tendency. These results demonstrated that high-salt diet impairs cardiac function, which can be attenuated by simultaneous administration of capsaicin through the impact on TRPV1.

### 3.3. Activation of TRPV1 by Dietary Capsaicin Attenuates Cardiac Hypertrophy and Fibrosis on Long-Term High-Salt Diet

**Figure 3:**
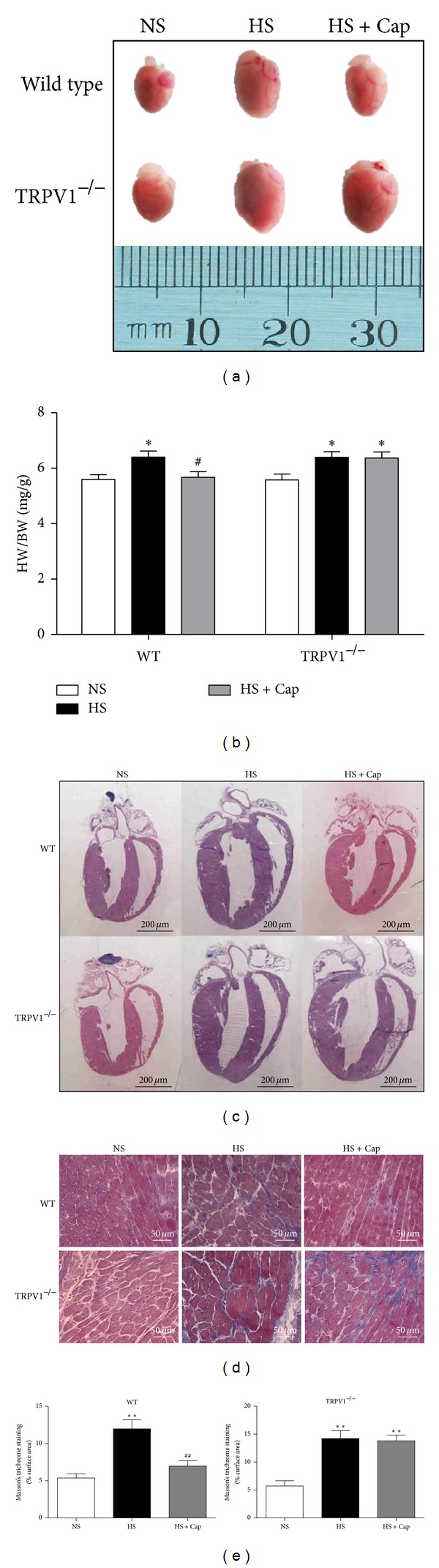
Dietary capsaicin attenuates cardiac hypertrophy and fibrosis in WT mice on long-term high-salt diet through TRPV1 activation. (a) Photographs of sample hearts from WT and TRPV1^−/−^ on NS, HS, and HS + Cap group. (b) Heart weight index from various groups (*n* = 7). Mean ± SEM: *P < 0.05 versus NS; ^#^ *P* < 0.05 versus HS. (c) Low-magnification view of hematoxylin and eosin stains of different group histological sections. (d) Masson's trichrome staining for collage deposition of histological sections from various groups. (e) Quantification of interstitial fibrotic area. Values were expressed as the mean ± SEM from 7 separate experiments. **P < 0.01 versus NS; ^##^ *P* < 0.01 versus HS.

At the end of the experimental period, the mice were subsequently killed, and the heart was excised. Consistent with the observations made by echocardiography, heart weight index was remarkably increased in both WT and TRPV1^−/−^ mice in the HS group compared to NS control group (Figure 3(a), both *P* < 0.05). Chronic capsaicin feeding markedly prevented high-salt-induced heart weight index increased in WT mice (*P* < 0.05), but no difference was seen in TRPV1-null mice. Hematoxylin and eosin stains of the heart demonstrated a marked hypertrophy after 1 year of HS diet compared to the control group (Figure 3(c)). Treatment with capsaicin attenuated the hypertrophy in WT mice fed with HS diet but not in TRPV1-null mice fed with the same diet. Long-term high-salt diet significantly increased collagen deposition as determined by Masson trichrome staining of the left ventricular sections compared to the control (normal-salt fed) group (Figure 3(d)). Chronic capsaicin feeding significantly suppressed high-salt-induced cardiac fibrosis, but no difference was seen in the TRPV1-null mice.

### 3.4. Activation of TRPV1 by Dietary Capsaicin Upregulates PPAR-*δ* and UCP2 Protein Expression and Decreases iNOS Production in Mice on Long-Term High-Salt Diet

**Figure 4:**
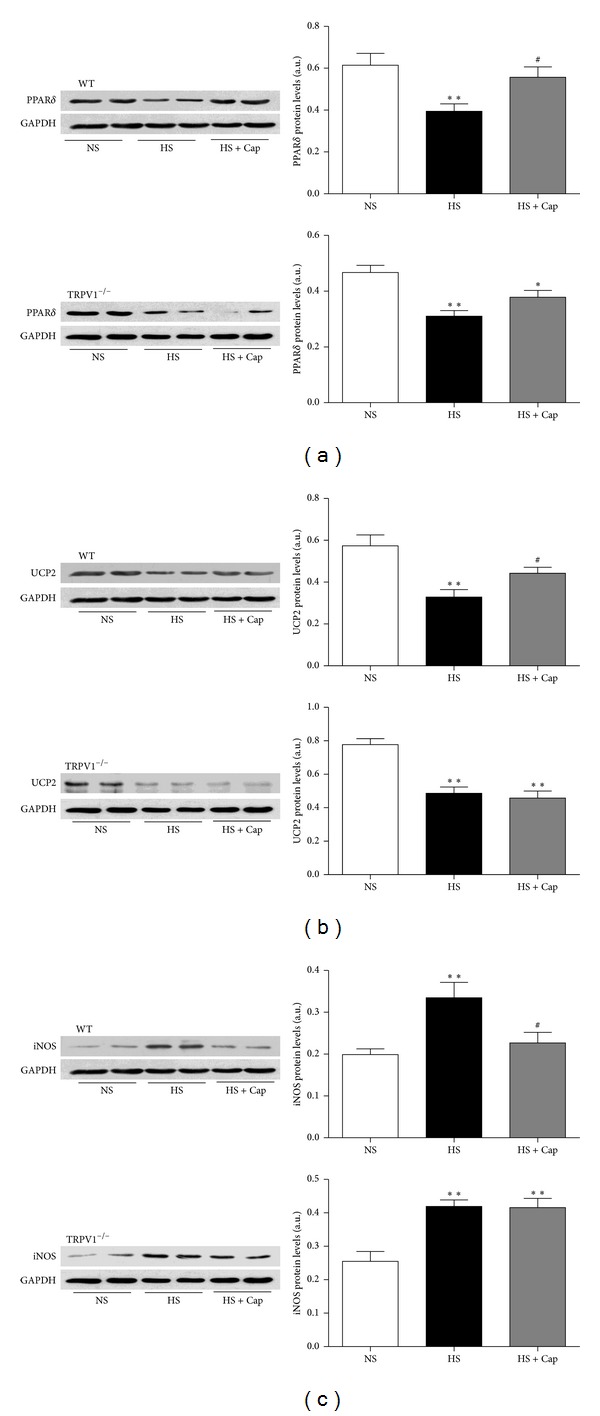
Dietary capsaicin upregulates PPAR-*δ* and UCP2 protein expression and decreases iNOS production in mice on long-term high-salt diet through TRPV1 activation. Immunoblottings of PPAR-*δ* (a), UCP2 (b), and iNOS (c) were performed in the left ventricular in WT and TRPV1^−/−^ from various groups. Data are means ± SEM from 3 separate experiments. *P < 0.05, **P < 0.01 versus NS, ^#^ *P* < 0.05 versus HS.

To explore the mechanism underlying capsaicin's action, we further examined the protein expression of left ventricular. As shown in Figure 4, the protein expression of PPAR-*δ* and UCP2 was decreased in both WT and TRPV1^−/−^ mice in the HS group compared to NS control group (both *P* < 0.05). Chronic capsaicin feeding caused significant upregulation of PPAR-*δ* and UCP2 in the left ventricular muscles of WT mice (*P* < 0.05), but not in those of TRPV1-null mice. However, protein expression of iNOS was increased in both WT and TRPV1^−/−^ mice in the HS group compared to NS control group, and chronic capsaicin feeding suppresses upregulation of iNOS in the left ventricular muscles of WT mice (*P* < 0.05), but not in those of TRPV1-null mice.

### 3.5. TRPV1 Activation by Capsaicin Increases PPAR-*δ* Expression in Cardiomyocytes

**Figure 5:**
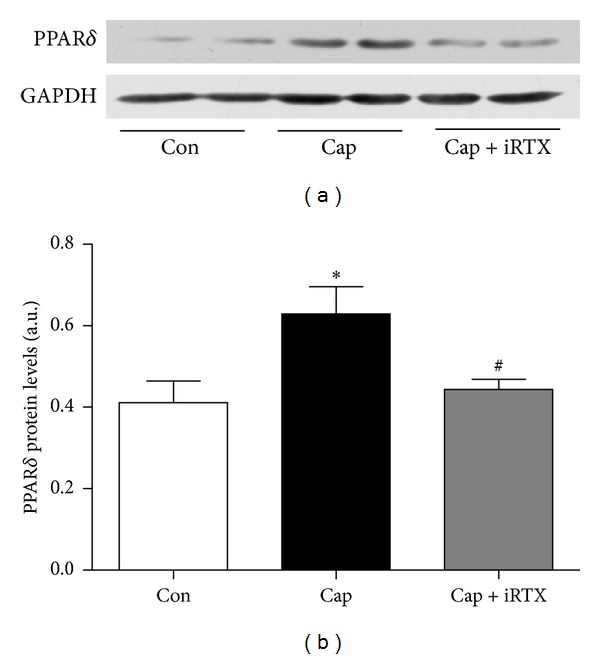
TRPV1 activation increases PPAR-*δ* expression in H9c2 cells. Immunoblotting of PPAR-*δ* was performed in H9c2 cardiomyocytes stimulated with capsaicin (1 *μ*mol/L) in the presence or absence of iRTX (1 *μ*mol/L) for 24 h. Data are means ± SEM from 3 separate experiments. *P < 0.05 versus control; ^#^ *P* < 0.05 versus capsaicin.

To better understand the possible molecular mechanisms for activation of TRPV1 by capsaicin which upregulates PPAR-*δ* expression, we detected the protein changes in the cultured H9c2 cells stimulated by capsaicin and iRTX. As shown in Figure 5, the protein expression of PPAR-*δ* was increased in the capsaicin group compared to control group (*P* < 0.01), which was partially reduced by iRTX treatment (*P* < 0.05). The finding further supports that capsaicin upregulates PPAR-*δ* through TRPV1 activation.

### 3.6. Activation of TRPV1 by Dietary Capsaicin Relieves Oxidative/Nitrotyrosine Stress in Mice on Long-Term High-Salt Diet

**Figure 6:**
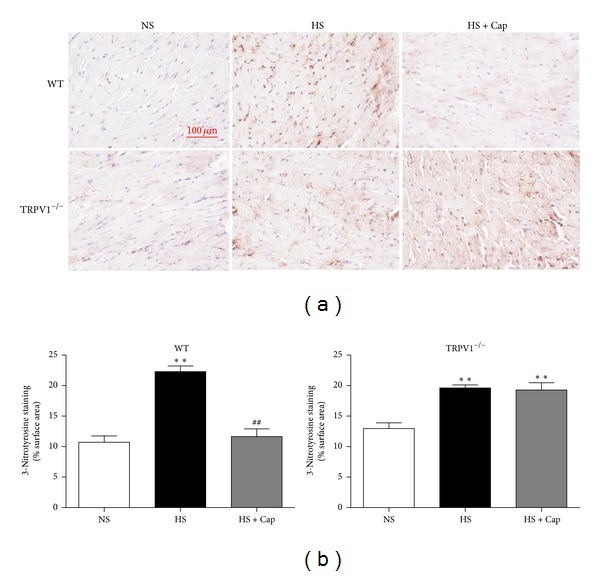
Activation of TRPV1 relieves oxidative/nitrotyrosine stress in mice on long-term high-salt diet. (a) Representative examples of LV sections from WT and TRPV1^−/−^ mice stained for 3-nitrotyrosine. (b) 3-Nitrotyrosine staining and scoring of expression in LV of various groups. Values were expressed as the mean ± SEM from 7 separate experiments. **P < 0.01 versus NS; ^##^ *P* < 0.01 versus HS.

As shown in Figure 6, left ventricular 3-nitrotyrosine levels, used to assess oxidative/nitrosative stress, were remarkably increased in both WT and TRPV1^−/−^ mice in HS group compared to NS control group (*P* < 0.01), and dietary capsaicin prevented the increase of 3-nitrotyrosine levels in WT mice on a high-salt diet (*P* < 0.01). However, this effect of capsaicin was absent in TRPV1^−/−^ mice on a high-salt diet. The finding further supports that activation of TRPV1 by dietary capsaicin relieves oxidative/nitrotyrosine stress caused by long-term high-salt diet.

## 4. Discussion

A number of experimental and clinical observations indicate that cardiometabolic diseases are closely related to unhealthy dietary history [16]. High-salt diet, which is common in most districts of China, may have harmful effects, for instance, causing a rise in blood pressure and increasing the risk of stroke, left ventricular hypertrophy, and renal disease [17]. Thus, dietary interventions become a popular recommendation for reducing the risk of cardiovascular disease. Major findings of the present study show that dietary capsaicin consumption attenuates high-salt-induced cardiac hypertrophy through TRPV1 activation in myocardium.

Most previous investigations pay more attention to TRPV1 that acted as a molecular integrator of multiple chemical and physical stimuli [18]. However, TRPV1 has recently been reported to play different role in various cells and tissues including adipocyte, skeletal muscle, vessel, and myocardium [19, 20]. TRPV1 plays a protective role in myocardial ischemia via preventing LV remodeling and cardiac functional deterioration, enhancing myocardium healing and regeneration. The present study demonstrates for the first time that chronic TRPV1 activation induced PPAR-*δ* expression to counteract high-salt-induced cardiac hypertrophy. Studies involving PPAR-*δ* demonstrate the importance of PPAR-*δ* in multiple biological processes related to cardiac development and function. For example, PPAR-*δ* activation exerts a protective role in atherosclerosis, myocardial injury, and cardiac hypertrophy [21–24]. Moreover, PPAR-*δ* alleviates cardiac fibrosis via suppressing cardiac fibroblast proliferation in rat neonatal cardiac fibroblast [25]. In our study, protein expression of PPAR-*δ* was decreased in both WT and TRPV1^−/−^ mice in the HS group, which eventually exhibited notable left ventricular hypertrophy. On the contrary, the given capsaicin caused a significant upregulation of PPAR-*δ* in the left ventricular muscles of WT mice, but not in those of TRPV1-null mice. In addition, we also discover that capsaicin exposure resulted in a remarkable increase in PPAR-*δ* expression in cultured H9c2 cells, which was blocked by the specific TRPV1 antagonist iRTX.

PPAR-*δ* activation is shown to induce UCP2 expression in several cell lines, such as H9c2 cells, adipose cells, and human muscle cells [26–28]. UCP2 is a widely expressed mitochondrial inner membrane carrier protein that regulates the mitochondrial membrane potential created by the proton gradient across the inner mitochondrial membrane [29]. UCP2 is also thought to play a role in the detoxification of ROS produced by the mitochondria given that mitochondrial production of ROS is dependent on the mitochondrial membrane potential [30, 31]. For example, the genetic absence of UCP2 exacerbates high-salt-induced vascular dysfunction because of enhanced oxidative stress in vessels [32], while overexpression of UCP2 is protective against mitochondrial death as a result of oxidative stress in cardiomyocyte [33]. Although UCP2 has been linked to oxidative stress, the role of UCP2 in heat of mice facing HS intake challenge is rarely elucidated. Our study demonstrated a decrease of UCP2 in the left ventricular of HS group, which was restored after TRPV1 activation. This finding appears to provide further evidence that the protective role of TRPV1 in myocardium could be related to UCP2 expression.

There are three nitric oxide synthase (NOS) isoforms in cardiomyocytes, between which inducible NOS (iNOS, NOS2) is absent in the healthy heart, and its expression is induced in response to stimuli such as tissue injury and inflammation [34]. Increased iNOS expression in myocardium is able to launch a process of cardiac remodeling which is characterized by cardiac hypertrophy and dilatation of cardiac chamber [35]. It has been reported that the increased iNOS induced by ischemia/reperfusion injury or endotoxic shock in myocardium can be attenuated through PPAR-*δ* activation, the mechanism of which may implicate the influence of PPAR-*δ* on transcriptional control of iNOS expression [36, 37]. Keeping with those findings, our studies indicate that long-term high-salt diet markedly increases the protein expression of iNOS and decreases the expression of PPAR-*δ*, while dietary capsaicin could reverse these effects. Other studies reveal that overexpression of iNOS in cardiac myocytes resulted in increased myocardial peroxynitrite, myocardial fibrosis, and ventricular hypertrophy, but ablation of iNOS shows less severe myocardial infarction-induced left ventricular remodeling compared to wild-type mice [38, 39].

The detrimental effect of iNOS expression could be due to producing excessive NO accompanied by increased production of ROS, including peroxynitrite and superoxide [40]. Nitrotyrosine formation, along with its detection by immunostaining, is originally proposed as a relatively specific marker for the detection of the endogenous formation of peroxynitrite [41]. Increased oxidative stress is considered to be a major determinant of protein nitration and peroxynitrite [42]. Thus, to further clarify cardiac structural damage caused by oxidative stress in HS-induced cardiac hypertrophy, the present study determines the level of oxidative stress by immunostaining with anti-nitrotyrosine antibody. We have seen an increase in nitrotyrosine staining in samples of HS group, which was restored after TRPV1 activation. This finding appears to provide further evidence that the protective role of TRPV1 in myocardium could be related to decreased iNOS production.

In conclusion, our studies demonstrate for the first time that chronic dietary capsaicin improves cardiac hypertrophy and fibrosis caused by long-term high-salt diet. This benefit effect is likely to be caused by TRPV1 mediated upregulation of PPAR-*δ* expression, which can protect heart from oxidative stress-induced myocardium damage. This protection in part upregulates the expression of UCP2 and suppresses the myocardial production of iNOS. Our findings show new insights into the potential role of TRPV1 channel in the regulation of high-salt-induced cardiac hypertrophy and fibrosis. TRPV1 channel activation via dietary capsaicin can be a promising lifestyle modification strategy in the reduction of the harmful effect of excessive salt intake.

## Figures and Tables

**Table 1:** Cardiac function from WT and TRPV1^−/−^ mice on ND, HS or HS + Cap diet for 1 year.

Parameter	WT			TRPV1^−/−^		
	ND	HS	HS + Cap	Nd	HS	HS + Cap
No.	8	8	8	8	8	8
BW, g	27.9 ± 4.2	27.5 ± 3.2	26.9 ± 3.8	30.7 ± 2.0	26.1 ± 2.3	26.2 ± 2.2
LVEDD, 10^−5^ m	233 ± 29	287 ± 36∗∗	245 ± 35^#^	219 ± 44	280 ± 36^†^	288 ± 50^†^
LVESD, 10^−5^ m	158 ± 27	211 ± 28∗∗	168 ± 22^##^	147 ± 32	208 ± 25^††^	218 ± 31^††^
LVPW, 10^−5^ m	79 ± 16	103 ± 16∗	75 ± 15^##^	72 ± 15	91 ± 12^†^	88 ± 9^†^
%FS	32 ± 4	26 ± 2∗∗	31 ± 5^#^	33 ± 6	26 ± 4^†^	24 ± 6^†^
LVW/BW (mg/g)	1.93 ± 0.51	3.56 ± 0.90∗∗	2.08 ± 0.85^##^	1.58 ± 0.32	2.95 ± 0.63^†^	2.93 ± 0.67^†^

Data are expressed as the mean ± SEM.

BW, body weight; FS, fractional shortening; LVEDD, LV end-diastolic diameter; LVESD, LV end-systolic diameter; LVPW, LV posterior wall; LVW, LV weight; WT wild-type mice; TRPV1^−/−^ TRPV1-null mice; NS normal-salt diet; HS high-salt diet; HS + Cap high-salt plus capsaicin diet; ∗*P* < 0.05  ∗∗*P* < 0.01 versus WT-NS; ^#^ *P* < 0.05  ^##^ *P* < 0.01 versus WT-HS; ^†^ *P* < 0.05  ^††^ *P* < 0.01 versus TRPV1^−/−^-NS.
